# SelectBCM tool: a batch evaluation framework to select the most appropriate batch-correction methods for bulk transcriptome analysis

**DOI:** 10.1093/nargab/lqad014

**Published:** 2023-03-03

**Authors:** Madhulika Mishra, Lucas Barck, Pablo Moreno, Guillaume Heger, Yuyao Song, Janet M Thornton, Irene Papatheodorou

**Affiliations:** European Molecular Biology Laboratory, European Bioinformatics Institute, The Wellcome Trust Genome Campus, Hinxton, Cambridge CB10 1SD, UK; Open Targets, Welcome Genome Campus, Hinxton, Cambridge CB10 1SD, UK; European Molecular Biology Laboratory, European Bioinformatics Institute, The Wellcome Trust Genome Campus, Hinxton, Cambridge CB10 1SD, UK; Open Targets, Welcome Genome Campus, Hinxton, Cambridge CB10 1SD, UK; Heidelberg University, Grabengasse 1, 69117 Heidelberg, Germany; European Molecular Biology Laboratory, European Bioinformatics Institute, The Wellcome Trust Genome Campus, Hinxton, Cambridge CB10 1SD, UK; Open Targets, Welcome Genome Campus, Hinxton, Cambridge CB10 1SD, UK; European Molecular Biology Laboratory, European Bioinformatics Institute, The Wellcome Trust Genome Campus, Hinxton, Cambridge CB10 1SD, UK; GSK, Gunnels Wood Road, Stevenage, Hertfordshire SG1 2NY, UK; European Molecular Biology Laboratory, European Bioinformatics Institute, The Wellcome Trust Genome Campus, Hinxton, Cambridge CB10 1SD, UK; European Molecular Biology Laboratory, European Bioinformatics Institute, The Wellcome Trust Genome Campus, Hinxton, Cambridge CB10 1SD, UK; Open Targets, Welcome Genome Campus, Hinxton, Cambridge CB10 1SD, UK; European Molecular Biology Laboratory, European Bioinformatics Institute, The Wellcome Trust Genome Campus, Hinxton, Cambridge CB10 1SD, UK; Open Targets, Welcome Genome Campus, Hinxton, Cambridge CB10 1SD, UK

## Abstract

Bulk transcriptomes are an essential data resource for understanding basic and disease biology. However, integrating information from different experiments remains challenging because of the batch effect generated by various technological and biological variations in the transcriptome. Numerous batch-correction methods to deal with this batch effect have been developed in the past. However, a user-friendly workflow to select the most appropriate batch-correction method for the given set of experiments is still missing. We present the SelectBCM tool that prioritizes the most appropriate batch-correction method for a given set of bulk transcriptomic experiments, improving biological clustering and gene differential expression analysis. We demonstrate the applicability of the SelectBCM tool on analyses of real data for two common diseases, rheumatoid arthritis and osteoarthritis, and one example to characterize a biological state, where we performed a meta-analysis of the macrophage activation state. The R package is available at https://github.com/ebi-gene-expression-group/selectBCM.

## INTRODUCTION

Over the last two decades, high-throughput transcriptome studies have resulted in a vast accumulation of bulk gene-expression experiments in public repositories and value-added resources, including microarray and RNA-seq data (1–3). The potential applications of accumulated transcriptomic datasets are numerous, such as unifying large-scale studies from the GTEx project and TCGA consortium to identify robust gene signatures (4–6). Another example is a multi-cohort transcriptomic analysis to identify conserved host immune responses across viruses (7). An emerging focus is to use available resources for cell-type decomposition for the bulk gene-expression experiments benefiting from both single-cell and bulk expression experiments, for example, characterizing cellular heterogeneity in resected tumour biopsies from patients with melanoma in the TCGA cohort using single-cell RNA-seq (scRNA-seq) reference data (8,9).

However, there are several challenges before fully harnessing this wealth of data for scientific investigation; one of the most critical is the ‘batch effect’ in collated transcriptome datasets (10,11). Batch effects capture non-biological confounding factors such as array design, experimental batches, etc. and often interfere with distinguishing biological signals from the integrated datasets (4,12). In the past few years, along with developing a method to integrate collated bulk gene-expression experiments, numerous batch-correction methods (BCMs) have evolved to address batch effects in the integrated datasets. For microarray technology, the most popular BCMs are ComBat (13) and limma (14) when the source of variation is known, and in the case of unknown variation, RUV (15). Similarly, RUVSeq (16) and ComBat-seq (17) are specially designed for bulk RNA-seq experiments; other widely used methods are ComBat and limma. However, the choice of BCM depends on the authors; often, there is no systematic approach to choosing the appropriate BCM. Even after achieving significant milestones in bulk gene-expression integration, a user-friendly workflow to select the best BCM for a given collection of datasets is missing.

Given the tremendous potential buried in the available bulk transcriptome datasets, we aimed to develop a freely available R-based tool to facilitate gene-expression integration. Here, we present the SelectBCM tool to choose the appropriate BCM for the given set of bulk transcriptomic data. Integrating different BCM tools and assessment strategies into the same framework, the SelectBCM tool, will increase the reusability and reproducibility of these BCMs for batch correction and allow the user to compare and select the top BCM for their chosen datasets. We also demonstrate the application of SelectBCM through case studies and compare differentially expressed genes (DEGs) obtained after batch correction with the traditional meta-analysis approach.

## MATERIALS AND METHODS

### Overview of implemented BCMs

We implemented a selection of the most widely used BCMs in our SelectBCM tool. We summarize the key characteristics of the nine BCMs implemented in Supplementary Data D1. For microarray technology, the workflow contains BCMs ranging from the traditional limma (14) to mnnCorrect (18), the latest technique for batch correction in single-cell transcriptomes. We implemented both empirical and non-empirical versions of ComBat (13), labelled ComBat1 and ComBat2, respectively, plus Q_ComBat, which performs quantile normalization before applying the batch correction with parametric adjustment of ComBat. We again implemented various flavours for the RUV (15), including the naiveRandRUV_HK, which uses literature-derived control genes for *Homo sapiens* (19), while the naiveRandRUV_empi.controls method empirically derives control genes from input data. We also include quantile combined naiveRandRUV versions in our workflow.

For RNA-seq experiments in SelectBCM, we include used BCMs developed for bulk RNA-seq and single-cell technologies. We include limma (14), ComBat (13) and RUVSeq/RUVs (16) implemented with the default settings. We implement null models (without knowledge of biological signal) and complete models (with knowledge of biological signal) of the ComBat-seq (17). Since ComBat-seq uses a more sophisticated negative binomial regression model to estimate batch effects on the count matrix in RNA-seq, we expect it to perform better than limma or ComBat. We then include mnnCorrect (18), a single-cell BCM, in our pipeline to test whether it can be applied to bulk transcriptome batch correction. However, it is important to note that mnnCorrect utilizes mutual nearest neighbours in contrast to surrogate variable-based methods to infer and correct batch effect. We also include scBatch (20) with the default settings, which performs batch-effect correction on both bulk RNA-seq and scRNA-seq data and is not restricted by assumptions on the mechanism of batch-effect generation.

### Evaluation methods

We incorporated a series of evaluation methods to measure how well each BCM implemented in the SelectBCM tool has performed in removing batch effects (summary provided in Supplementary Data D2). Below we describe each of the assessment methods in detail.

#### Principal variance component analysis

We implemented the principal variance component analysis (PVCA) tool to estimate the batch effect and biological variability of interest in the SelectBCM tool in a given meta-experiment (21). In summary, PVCA first performs principal component analysis (PCA) and then conducts variance component analysis to fit a mixed linear model using variability sources (e.g. batch or biological factors, such as age, gender or disease) as random effects to estimate and partition the total variability.

#### Silhouette coefficient

To assess the clustering pattern of biosamples before and after batch correction, we compute the silhouette coefficient (18). Theoretically, the silhouette score *s*(*i*) can range from –1 to 1, with ${s}( i ) \to - 1$ if two clusters are separate and $s( i ) \to - 1$ if two clusters overlap but have dissimilar variance (i.e. significant batch effect). If $( i ) \to 0$, both groups have roughly the same structure (i.e. negligible batch effect). Thus, we use the absolute value *s* as an indicator of the presence or absence of ‘batch effects’.

#### pcRegression

To estimate the linear batch effect, we perform linear regression using the top 20 principal components (PCs) obtained from PCA as a default setting against a batch (categorical) variable. For the estimation purpose, we use the scaled pcRegression score by scaling it by the total variance of the top 20 PCs, as suggested in the κBET package (22).

#### Entropy

We compute the regional entropy of batch mixing before and after batch correction using the first two PCs, as indicated in the MNN package (18). In our workflow, users can vary the input number of biosamples to compute entropy depending on the size of their meta-experiment.

#### Highly variable genes

Highly variable genes (HVGs) contribute to cell-to-cell variation within a homogeneous cell population and represent true biological heterogeneity (23,24). We qualitatively measure the impact of the batch correction on biological heterogeneity by measuring the preservation of HVG after applying BCMs. We devised HVG.union, where we compute a ratio of the number of conserved HVGs after batch correction to the set union of HVGs derived from individual experiments and use it as a qualitative measure for biological heterogeneity.

### Ranking of BCMs

**Figure 1.**
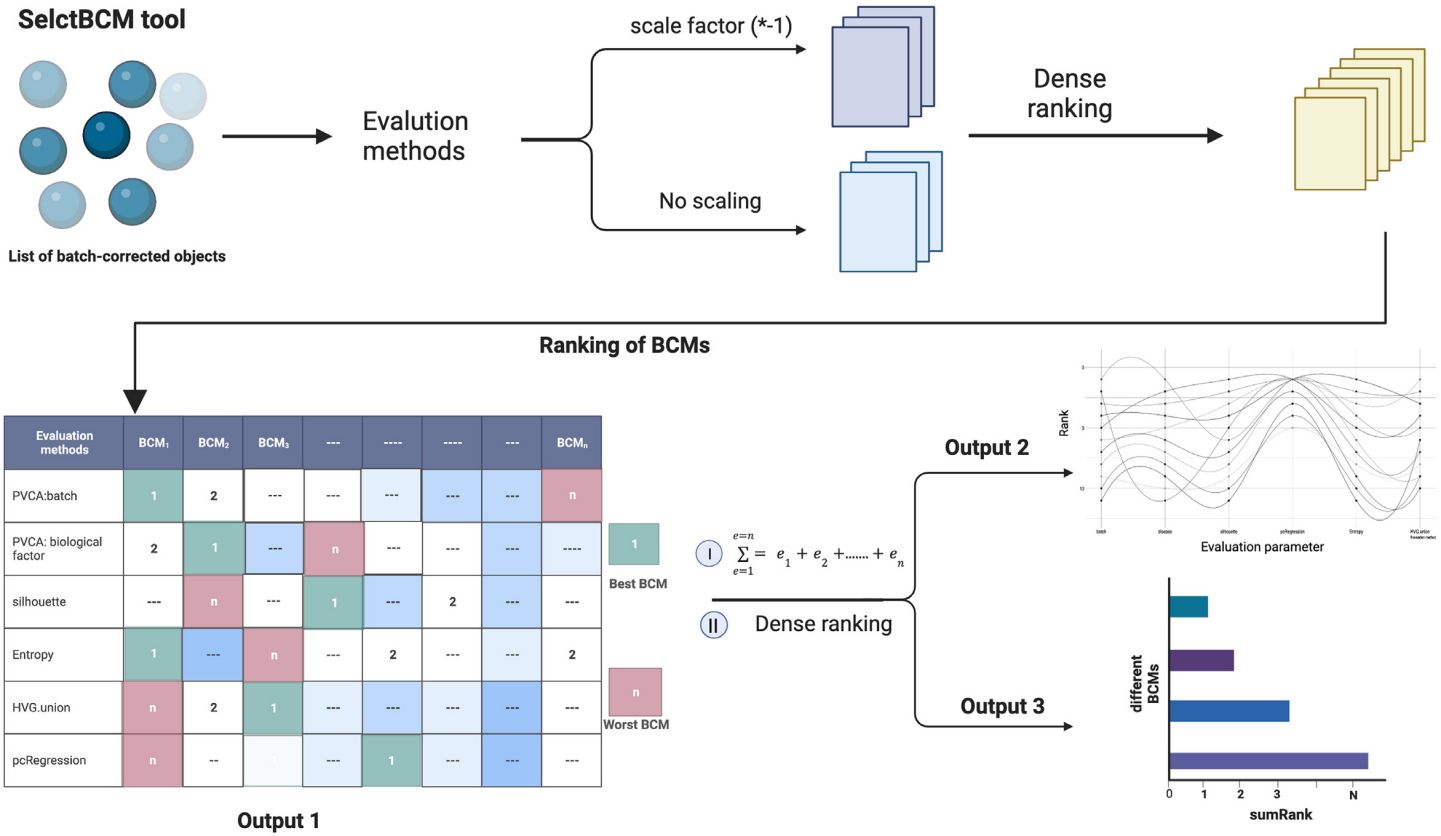
Diagrammatic schema for ranking of BCMs. SelectBCM first uses various evaluation methods to test the performance of BCMs. To make scoring consistent, where the first rank always means the best performer, SelectBCM first performs data transformation on PVCA: batch, silhouette index and pcRegression output obtained from the evaluation step, and then dense ranking for each output. This step results in an evaluation matrix (Output 1) containing the ranking of each BCM for the given evaluation method. Using Output 1, SelectBCM provides a diagnostic plot showing the performance of BCMs across evaluation methods and the final rank plot of BCMs where the first rank represents the most appropriate BCM for the given set of experiments.

To rank BCMs, we compute sumRank using results obtained from evaluation steps as parameters (Figure 1). In the ranking step, we first perform data transformations on results obtained from the evaluation step and transform them into an evaluation matrix (Output 1). We describe data transformation steps below:

- PVCA: batch (${e_1}$), silhouette index (${e_2})$ and pcRegression (${e_3})$ first get scaled by multiplying by −1, and then ordered using the dense ranking method. This ensures that BCM with rank 1 denotes the best BCM, as all three methods present batch effect in the data.
- PVCA: biological factor (${e_4}$), entropy (${e_5})$ and HVG.union (${e_6})$ are directly dense ranked indicating the first ranked method as the best method.

We provide an evaluation matrix as a diagnostic plot (Output 2) for visualization purposes. We then compute sumRank using the evaluation matrix explained below:


$$\begin{equation*}{\rm{sumRank}}= {\rm{dense}}\,{\rm{rank}}\left( {\sum_{{{e = 1}}}^{{{e = 6}}} {{{{e}}_{\rm{1}}}{{ + }}{{{e}}_{\rm{2}}}{{ + \cdots + }}{{{e}}_{{6}}}} } \right),\end{equation*}$$

where $e$ denotes evaluation parameters present in the evaluation matrix.

Therefore, sumRank provides the final ranking of BCMs for the given meta-experiment in descending order. BCM ranked higher across evaluation methods will get a higher weightage and, therefore, first rank among others (Output 3).

### Impact of top-ranked BCMs on biological inferences

We used visual and DEG analysis approaches to qualitatively assess the impact of the top 3 BCMs on biological inferences. To visually assess the impact of batch correction on biological clustering, we applied PCA on a meta-experiment obtained after batch correction and compared how well biosamples from different batches were mixed. We perform DEG analysis to assess the impact of the batch correction on identifying relevant biological signals. We also compare DEGs identified by BCMs with those determined by the traditional meta-analysis approach (MetaVolcanoR), where people study each experiment individually and then statistically combine the list of significant genes (25). We provide a detailed description of DEG analysis and biological enrichment analysis (Supplementary Text S1). We also describe MetaVolcanoR and the differences between MetaVolcanoR and current approaches in Supplementary Text S2 and Supplementary Figure S1.

## RESULTS

### SelectBCM tool

**Figure 2.**
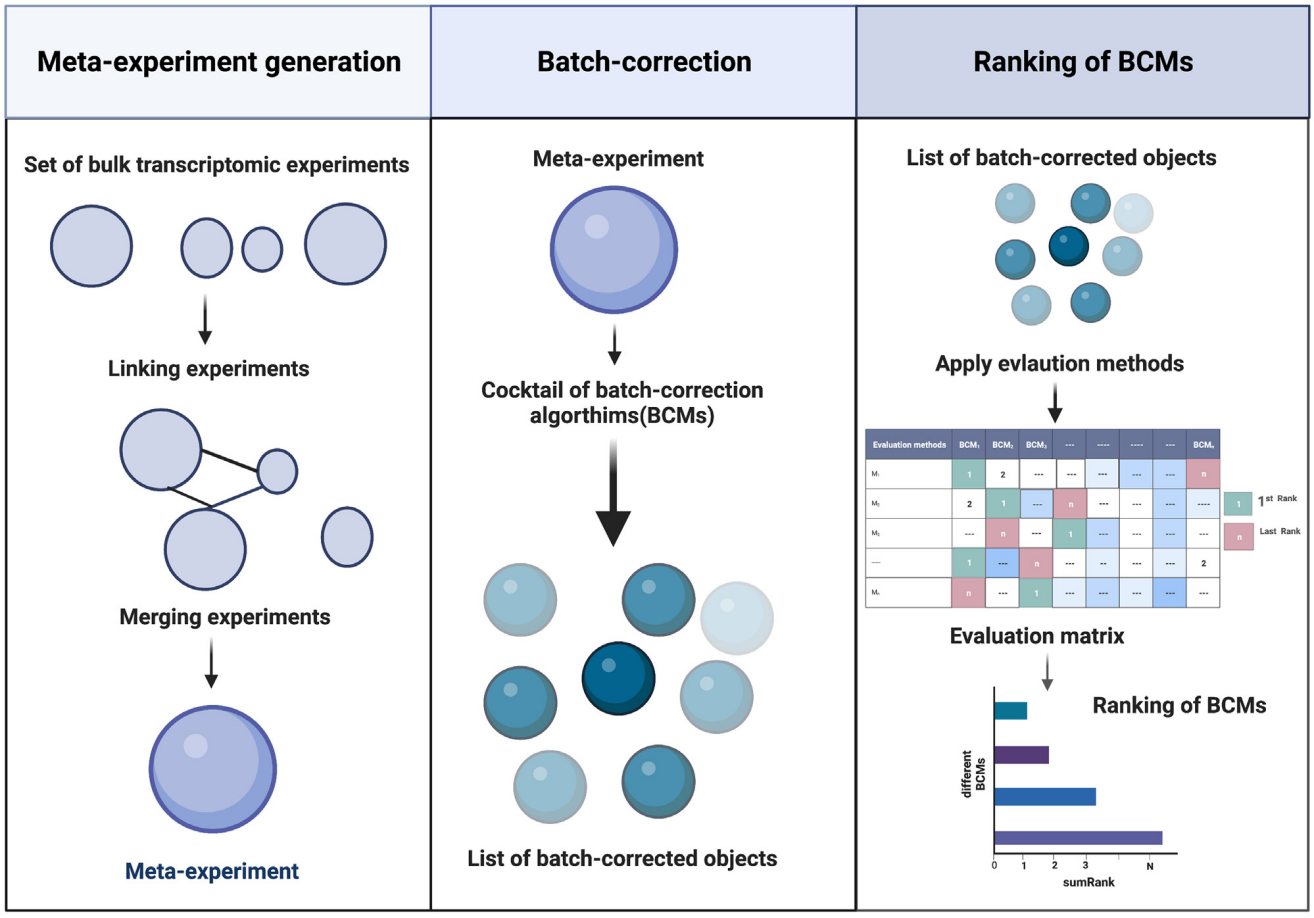
Overview of the SelectBCM tool. SelectBCM takes experiments (the size of the circle denotes the number of biosamples) as input and connects them through common biological attributes. Any unlinked experiment gets filtered out at this step. In the next step, the tool generates a meta-experiment by merging linked nodes. SelectBCM then performs batch correction on the meta-experiment using various BCMs and generates the output of BCM-corrected data as a list. In the later stage, SelectBCM applies different evaluation methods to test the performance of BCMs and the output rank of BCMs for the given meta-experiment.

The SelectBCM tool (https://github.com/ebi-gene-expression-group/selectBCM) implements a flexible framework in R to select the best BCMs for transcriptomic experiments (Figure 2). SelectBCM requires ExpressionSet (microarray experiment) or SummarizedExperiment (bulk RNA-seq experiment) as an input. This object must contain a log-normalized expression matrix for microarray or raw count matrix for bulk RNA-seq experiments and the relevant biological annotation of their biosamples in the object metadata. Since the workflow does not perform any quality check (QC), we strongly recommend that users perform QC for individual datasets before providing them as input. Each input dataset is then sent to the SelectBCM tool to undergo meta-experiment creation, batch correction, assessment and evaluation to prioritize BCMs. We describe input and available modules in SelectBCM in detail in Supplementary Text S3. At the end of the pipeline, users obtain a diagnostic plot reflecting how BCMs have performed across different evaluation methods, a final ranking list of BCM and the output of batch-corrected meta-experiment in the form of an R ‘RDS object’. These batch-corrected meta-experiments serve as input for a series of downstream analyses: (a) PCAs for visualization of the different batch corrections, (b) differential gene analysis, and (c) gene co-expression analysis and any other use cases for the user.

### Application to rheumatoid arthritis and osteoarthritis

We choose a meta-analysis of the common rheumatoid arthritis (RA) and osteoarthritis (OA) diseases as examples to demonstrate the usability of SelectBCM for microarray experiments. We curated microarray experiments for RA and OA from synovial fluid or bone specimens for *H. sapiens* from the widely used platforms Agilent and Affymetrix. We summarize the chosen datasets in Supplementary Data D3. We downloaded raw data from ArrayExpress (2), performed platform-appropriate pre-processing and converted probe IDs to Entrez IDs to make the study comparable. We consider each experiment as one batch, and the disease type is the biological variability we look for in the merged meta-experiment. We describe the results for RA disease in detail below.

#### RA case study

**Figure 3.**
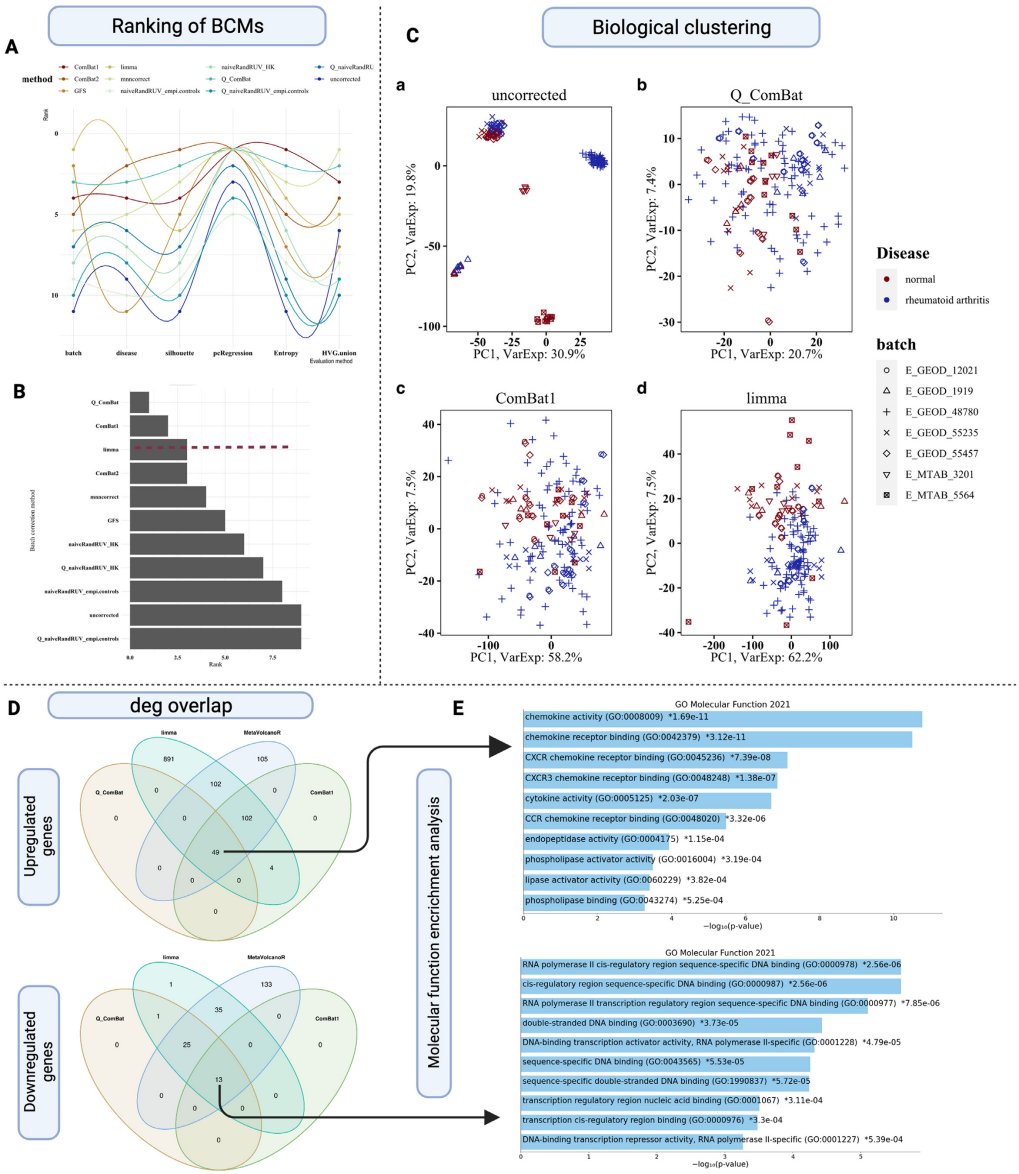
Meta-analysis of RA. (**A**) The diagnostic plot demonstrates that the performance of individual BCM is inconsistent among different evaluation methods. (**B**) Bar plot of sumRank provided by SelectBCM ranks Q_ComBat, ComBat and limma as top BCMs for the RA meta-experiment. (**C**) Performing batch correction improves biological clustering demonstrated in the PCA plot; biosamples are coloured by disease class and shaped by batch. (**D**) Venn diagram of the overlap of upregulated and downregulated genes among methods. (**E**) GO molecular function enrichment analysis of common upregulated and downregulated genes shows that all methods could capture disease biology.

For RA disease, we selected E-GEOD-1919, E-GEOD-55235, E-GEOD-48780, E-GEOD-12021, E-GEOD-55457 and E-MTAB-3201 experiments contributing at least RA or control synovial specimen samples or both. SelectBCM performs batch correction and evaluation for the RA meta-experiment. We observe that the evaluation methods did not produce a consensus among themselves to identify the best BCM (Figure 3A, Supplementary Data D4). Even though PVCA, silhouette, pcRegression and entropy use PCs as input, they resulted in a different ranking of the BCMs. pcRegression seems least sensitive to BCM and therefore fails to discriminate between BCMs. In terms of preserving biological information, PVCA suggested limma, ComBat2 and Q_ComBat as the top 3 BCMs using disease parameters, while HVG.union choose mnnCorrect, Q_ComBat and ComBat. However, mnnCorrect is ranked poor by other evaluation methods because of the remnant batch effect. SelectBCM ranked Q_ComBat first as it ranks higher by most evaluation methods, followed by limma and ComBat1 (Figure 3B).

To assess the impact of the top 3 BCMs shortlisted by the SelectBCM on biological clustering, we carried out a PCA (Figure 3C). We first observe that batch correction by all three BCMs in the RA study improves the batch mixing after batch correction compared to uncorrected ones. In the case of uncorrected/quantile normalized meta-experiment, biosamples clustered according to batch rather than their biological attributes. In the case of Q_ComBat and limma, the disease class mainly drives groupings. However, in ComBat1, even though batch clusters aggregated, disease-class clustering only improved a little.

We assess the impact of the batch correction on differential gene analysis by comparing DEGs obtained after applying the top 3 BCMs with DEGs obtained from the traditional meta-analysis approach. As expected, different BCMs resulted in different sets of DEGs having minimal overlap (Q_ComBat:88, ComBat1:168 and limma:1123; we explain the discrepancy in DEGs’ number in detail in Supplementary Figure S2).

For the MetaVolcanoR approach, we excluded E-GEOD-48780, E-MTAB-3201 and E-MTAB-5564 because of a lack of control and disease biosamples in respective datasets and obtained 564 DEGs. Across all methods, we got minimal consensus (49 upregulated and 13 downregulated DEGs) (Figure 3D). We also observed that even though Q_ComBat captured a small number of DEGs, it had maximum overlap with other methods.

Next, we investigated molecular function associated with common upregulated (49 genes) and downregulated (13 genes) using the EnrichR tool (26). The top common upregulated enriched molecular processes were chemokine activity, chemokine receptor binding, CXCR receptor binding, cytokine activity and phospholipase activity and are directly relevant to RA disease biology (Figure 3E) (27–29). Double-stranded methylated DNA binding, *cis*-regulatory region sequence-specific DNA binding, RNA polymerase II transcription regulatory region sequence-specific DNA binding and double-stranded DNA binding were the top 5 downregulated molecular functions among top BCMs and MetaVolcanoR (Figure 3E) (30). Even though Q_ComBat produced the smallest set of DEGs, we observed that it could capture meaningful molecular processes.

Finally, we also checked the tissue specificity of these common DEGs using the Jensen tissue score from EnrichR (26). Jensen score is the literature-mined gene–tissue associations in humans, mice, rats and pigs calculated by integrating multiple sources of evidence: transcriptomics, proteomics (human only) and literature. We observed that commonly upregulated genes were associated with the immune system, stromal cell, natural killer (NK) cell lines, synovial tissue and mast cell (Supplementary Figure S3). In RA disease, the synovial joint becomes highly inflamed. The synovial microenvironment consists of recruited immune cells such as NK cells, mast cells and stromal cells with synovial tissue (31), indicating that upregulated common genes could retain tissue-specific information even after batch correction.

Based on biological clustering, DEG recovery, molecular functions and tissue specificity of common DEGs, we concluded that the top 3 BCMs could capture biologically relevant processes while retaining tissue-specific information for the RA study. However, depending on the choice of BCM, some methods (such as limma) will require a more stringent statistical cut-off to filter DEGs.

#### OA study

We performed a similar analysis for OA and provided a detailed study description in Supplementary Text S4, Supplementary Data D5 and Supplementary Figure S4. In summary, ComBat remains on the list of top 3 BCMs (ComBat2, Q_ComBat and ComBat1) and recovered relevant biological signals regarding DEGs, associated molecular functions and tissue specificity.

### Application to a meta-analysis of the macrophage activation state

**Figure 4.**
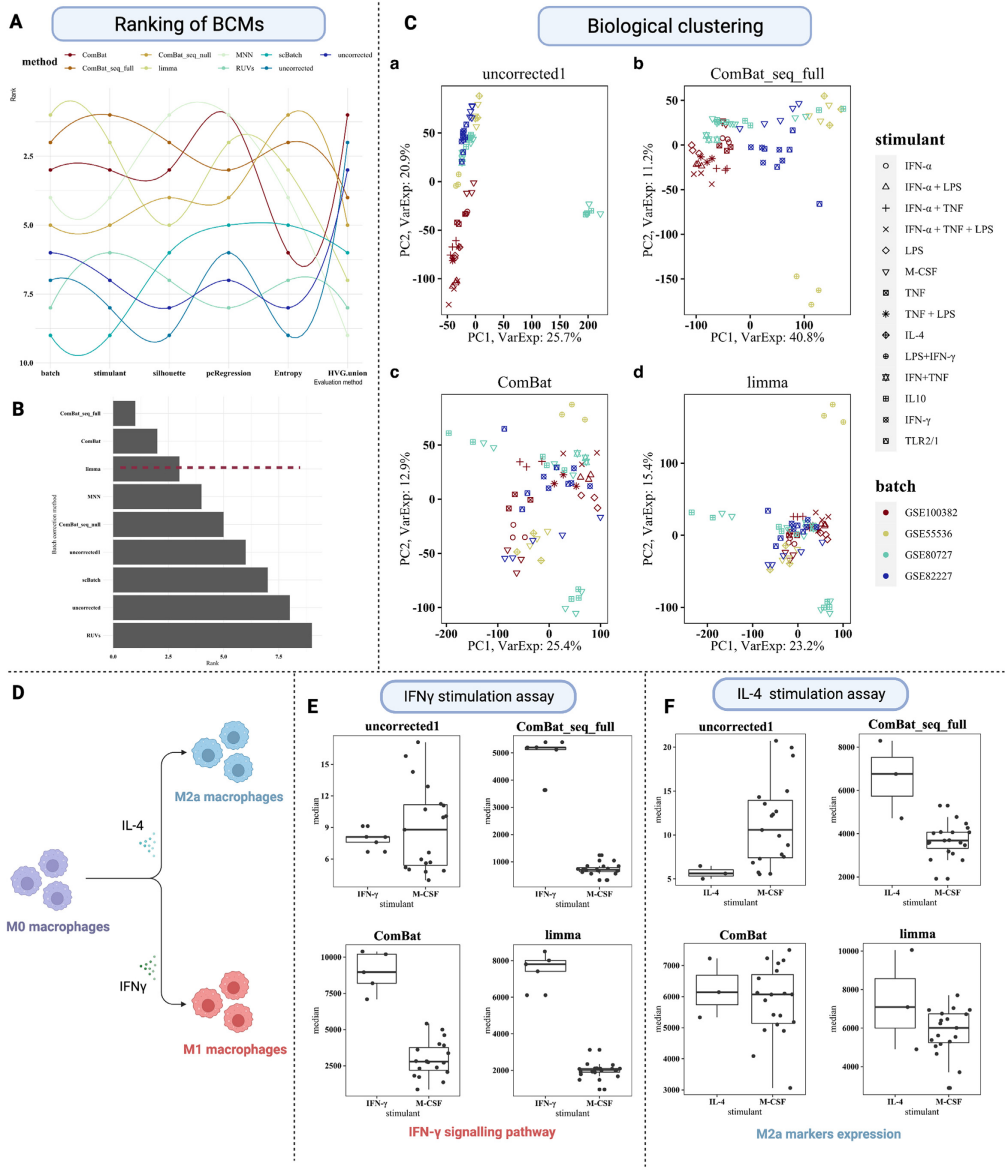
Meta-analysis of the MDM stimulation assay. (**A**) Performance of individual BCM based on different evaluation methods. (**B**) The SelectBCM tool chooses ComBat, ComBat_seq_full and limma as the top 3 BCMs for the MDM assay. (**C**) We observed that the biological clustering of biosamples did not improve even after batch correction; biosamples are coloured by batch and shaped by a different stimulant. (**D**) Schema of chosen phenotypes from the *in vitro* MDM study. We choose an interferon-gamma (IFN-γ) stimulant for the M1 phenotype representing pro-inflammatory macrophages and an interleukin-4 (IL-4) stimulant for the M2a phenotype, which drives tissue-repair mechanisms. (**E**) In the case of IFN-γ stimulation, all three BCMs capture the upregulation of M1 markers. (**F**) M2a markers are also captured upregulated upon IL-4 stimulation but failed to capture in uncorrected data. *Note*: Uncorrected1 is the variance stabilizing transformation-corrected meta-experiment.

We choose a meta-analysis of *in vitro* monocyte-derived macrophage (MDM) activation state models as a case study to demonstrate the application of the SelectBCM tool for bulk RNA-seq experiments. We choose the MDM study as macrophages represent heterogeneous immune cell populations in the context of tissue and diseases, and most studies use *in vitro* MDMs to understand the stimulant-associated heterogeneity (32–34). We selected GSE100382 (35), GSE55536 (36), GSE80727 (37) and GSE82227 (38) studies to perform a meta-analysis of *in vitro* MDM activation states. We summarize macrophage activation phenotype and associated stimulant for the current study design in Supplementary Text S5. We integrated datasets using the SelectBCM tool and performed batch correction and evaluation. We observed that except for HVG.union, other evaluation methods had consensus this time compared to previous examples (Figure 4A, Supplementary Data D6). Based on sumRank, SelectBCM chose ComBat, ComBatseq_full and limma as the top 3 BCMs for the *in vitro* MDM meta-experiment (Figure 4B).

We observed that though all three methods could remove batch effect (Figure 4C), the biological clustering of stimulants did not improve much because of inherent biological heterogeneity (39). We then identified DEG and associated pathways by comparing activated with unstimulated macrophages using DESeq2 (40). We choose two phenotypes, IFN-γ-induced M1 phenotype (drives pro-inflammatory immune response) and IL-4-induced M2a phenotype (drives tissue-repair mechanism), for downstream analysis (Figure 4D).

In the case of IFN-γ-induced M1 macrophages, we first checked expression changes in the IFN-γ signalling pathway as they are expected to be upregulated upon IFN-γ exposure. We observed that all three BCMs captured the upregulation of the IFN-γ response compared to the uncorrected meta-experiment (Figure 4E). Among the top 3 BCMs, we found 113 common DEGs, while none were in the uncorrected meta-experiment (Supplementary Figure S5). As expected, these genes mainly belong to interferon response-related pathways such as IFN-γ response, IFN-α response and the complement system.

In the case of the M2a phenotype (IL-4 stimulation assay), we again observed that all three BCMs captured the right direction of differential expression changes of marker genes of the IL-4 stimulation but failed to capture it in the uncorrected meta-experiment (Figure 4F). However, there was little consensus among the three BCMs in terms of identified DEGs (uncorrected1:0, ComBat_seq_full:301, limma:147 and ComBat: 24) (Supplementary Figure S5). Only ComBat_seq_full captured disease-relevant biological processes such as epithelial–mesenchymal transition, inflammatory response, TGF-β signalling and IL-6/JAK/STAT3 signalling (41–43). Therefore, we concluded that even though all three BCMs performed well in identifying stimulant-relevant marker gene-expression change, ComBat_seq_full is a better choice as BCM for the downstream biological analysis.

## CHALLENGES AND LIMITATIONS

Below we discuss the key challenges and limitations of performing batch correction in the bulk transcriptome. This study has emphasized the challenges associated with how biological heterogeneity interferes with the batch-correction outcome. Through a meta-analysis study of systemic juvenile idiopathic arthritis (neutrophil and blood transcriptome analysed separately), we demonstrate a scenario where biological heterogeneity (active and inactive states of illness) is present, the study design is unbalanced and the sample size is small. We observe that clustering did not improve after batch correction in both studies (Supplementary Data D7A and B, and Supplementary Figures S6 and S7). We also failed to identify any significant genes associated with the disease in both cases. We, therefore, recommend that users evaluate the importance of biological heterogeneity while performing batch correction, as suggested by many others in the field (10). Biological knowledge about the system’s understudies, such as relevant upregulated or downregulated pathways or marker genes, further helps select the most appropriate BCM from the top 3 for downstream analysis. In our experience, the study should be limited only to the comparable probe-size array/platforms in the case of microarray experiments. For example, having experiments from the old Affymetrix platform will limit the matching gene pool to a large extent (Supplementary Figure S8) and, therefore, should be avoided beforehand. Currently, SelectBCM can agnostically handle various species for data coming from bulk RNA-seq transcriptome but is limited to human transcriptome in the case of microarray.

## DISCUSSION

We designed the SelectBCM tool to solve the old problem of selecting the suitable BCM for bulk transcriptomic experiments. We implemented >9 state-of-the-art batch-correction algorithms in our workflow designed to handle bulk transcriptomes from microarray and RNA-seq technology. We showcase the useability of our workflow to address the problem of choosing the most appropriate BCM and the importance of having multiple evaluation methods. Using RA and OA datasets, we observed that Q_ComBat and ComBat1 were the top BCMs for better DEG recovery, and downstream biological inferences agreed with the traditional meta-analysis approach. For the bulk RNA-seq experiment, we observed that ComBat_seq is better for retaining biological signals. We expect our tool will speed up the bulk transcriptomic data integration process, starting from filtering the datasets based on metadata to batch correction and allowing users to focus on deciphering biological signals.

## DATA AVAILABILITY

The docker image of the tool is available at https://hub.docker.com/r/yysong123/selectbcm. All datasets and analyses used in the paper are public, referenced and downloadable at https://doi.org/10.5281/zenodo.7482141.

## Supplementary Material

lqad014_Supplemental_File
