# Correction to ‘SelectBCM tool: a batch evaluation framework to select the most appropriate batch-correction methods for bulk transcriptome analysis’

**DOI:** 10.1093/nargab/lqad027

**Published:** 2023-03-15

*NAR Genomics and Bioinformatics*, Volume 5, Issue 1, March 2023, lqad014, https://doi.org/10.1093/nargab/lqad014

In the originally published online version of this manuscript, there were errors within the affiliations for the authors.

For MM second affiliation should read: ‘Open Targets, Welcome Genome Campus, Hinxton, Cambridge CB10 1SD, UK’ instead of: ‘GSK, Gunnels Wood Road, Stevenage, Hertfordshire SG1 2NY, UK’

For LB third afffiliation should read: ‘Heidelberg University, Grabengasse 1, 69117 Heidelberg, Germany’ instead of: ‘GSK, Gunnels Wood Road, Stevenage, Hertfordshire SG1 2NY, UK’

For PM second affiliation should read: ‘Open Targets, Welcome Genome Campus, Hinxton, Cambridge CB10 1SD, UK’ instead of: ‘GSK, Gunnels Wood Road, Stevenage, Hertfordshire SG1 2NY, UK’

For GH second affiliation should read: ‘GSK, Gunnels Wood Road, Stevenage, Hertfordshire SG1 2NY, UK’ instead of: ‘Heidelberg University, Grabengasse 1, 69117 Heidelberg, Germany’

For JT second affiliation should read: ‘Open Targets, Welcome Genome Campus, Hinxton, Cambridge CB10 1SD, UK’ instead of: ‘GSK, Gunnels Wood Road, Stevenage, Hertfordshire SG1 2NY, UK’

For IP second affiliation should read: ‘Open Targets, Welcome Genome Campus, Hinxton, Cambridge CB10 1SD, UK’ instead of: ‘GSK, Gunnels Wood Road, Stevenage, Hertfordshire SG1 2NY, UK’

These errors have been corrected in the online version of the article.

