# Supplementary material for: SelectBCM tool: a batch evaluation framework to select the most appropriate batch-correction methods for bulk transcriptome analysis
Source: NAR Genom Bioinform. 2023 Mar 3;5(1):lqad014. doi: 10.1093/nargab/lqad014 (PMC9985330; doi:10.1093/nargab/lqad014)
Supplement: lqad014_Supplemental_File [file lqad014_supplemental_file.docx]

**SelectBCM Tool: A batch evaluation framework to select the most-appropriate batch-correction methods for bulk transcriptome analysis**

**Madhulika Mishra^1,4^, Lucas Barck^1,2,4^, Pablo Moreno^1,4^, Guillaume Heger^1,3^, Yuyao Song^1^, Janet M. Thornton^1,4^, Irene Papatheodorou^1,4^**

**Affiliations:**

**1** European Molecular Biology Laboratory, European Bioinformatics Institutes, The Wellcome Trust Genome Campus, Hinxton, Cambridge, United Kingdom, CB10 1 SD

**2** Heidelberg University, Grabengasse 1, 69117, Heidelberg, Germany

**3**  GSK, Gunnels Wood Road, Stevenage, Hertfordshire, United Kingdom, SG1 2NY

**4** Open Targets, Welcome Genome Campus, Hinxton, Cambridge, United Kingdom, CB10 1 SD

**supplementary text S1. A detailed description of differential gene analysis and biological enrichment analysis**

We perform deg analysis using limma for microarray experiments and DESeq2 package for bulk RNAseq experiments. In the case of microarray experiments, We applied Benjamini & Hochberg to correct for multiple testing and adjusted the *p*-value <0.05 and used fold change (FC) in expression as a variable filter (cutoff) to select genes as differentially expressed genes (degs)[(1)](https://paperpile.com/c/qCQuVv/4yqd). For the RNAseq experiment, we performed a one-way analysis of variance (ANOVA) to compare the change in the expression of genes between the control versus the rest of the other treatment conditions. We apply log2 fold change cutoff of more than one and q value cutoff <- 0.01 filter differentially expressed genes.

We performed GO molecular function enrichment analysis and Jensen tissue enrichment using EnrichR[(2–4)](https://paperpile.com/c/qCQuVv/mucc+zrKV+1FBl). For GO molecular function analysis, we use the GO molecular function 2021 as a resource and apply an adjusted p-value cut-off of 0.05 to identify significantly enriched processes. For tissue-enrichment analysis, we again apply the filter of adjusted p-value cut-off of 0.05 to identify significantly enriched tissue type.

**supplementary text S2.MetaVolcanoR approach for meta-analysis**

MetaVolcanoR aims at identifying “genes having consistent expression change across several studies”[(5, 6)](https://paperpile.com/c/qCQuVv/VAcA+jOfq). We implement the random effect model to identify genes altered in more than 50% of the datasets with a randomP (p-value) cut-off of <0.05. We performed MetaVolcanoR only in the microarray case studies.

**supplementary text S3.Detail description of selectBCM tool**

We compartmentalised the entire **Batchevaluation framework/selectBCM tool** into two main compartments. We describe the entire workflow in detail below.

We first describe the type of input the selectBCM tool can handle - it can take both microarray and bulk RNAseq transcriptomic data as input. For the microarray experiments, the tool takes ExpressionSet as an input. Each experiment should undergo preprocessing steps such as chip-appropriate background correction, probe to gene-level mapping, and log transformation in the expression matrix before starting the analysis. We also recommend removing low-expressed genes from the expression matrix. For bulk RNAseq experiments, input is standardised SummarizedExperiment with the gene-expression matrix as count matrix. selectBCM can also import data directly from the expression atlas as well as can load expression data from a local directory as well. We discourage people from mixing microarray and bulk RNAseq samples for statistical reasons.

In the first compartment of a framework, we create a meta-experiment from the given set of transcriptomic experiments. We first devised a computationally efficient logical step, “**remove_isolated_experiments**”, to create a meta-experiment using information solely present in the phenodata from the set of given transcriptomic experiments. This step is necessary because one must consider biological characteristics such as tissue origin and cell line of biosamples before accounting for batch effect. We use igraph library-based functions to link different experiments, making it handy for various other applications. It treats each experiment as one node and only connects it with others if both nodes share common biological attributes from information present in phenodata. The next step, “**merge_experiments**", merges linked experiments obtained from the previous step and generates one meta-experiment with an additional “batch” column in phenodata, where the batch represents one experiment.

The second compartment of the framework deals with batch correction of input meta-experiment and assessing and prioritising the best-performing BCM. We implemented widely used BCMs to remove the batch effect in the merged meta-experiment. In **Supplementary Data D1,** we summarise each batch-correction method - their original application, the required input and output formats generated. We have kept this step flexible enough to accommodate more upcoming BCMs if they arise. The output of the “**batch_correction**” step is the collection list of batch-corrected meta-experiment.

We then implemented the “**batch_evaluation**” step containing a cocktail of evaluation methods in our workflow to evaluate the performance of BCMs. Implemented methods are mainly principal components(PC) analysis-based. For example, PVCA, silhouette index, and PcRegression measure residual batch-effect in corrected data using top principal components. Therefore, the lower the score of these methods, the better the BCM performance.

On the contrary, well-mixed batches will have higher entropy; therefore, the best BCM will have the highest entropy score. We expect an excellent BCM to retain an actual biological signal and heterogeneity while removing the technical noise from the meta-experiment. HVG.union captures inherent biological heterogeneity for the given mixture and helps us identify over-fitting issues in batch-corrected data. For HVGs, the higher the rank of BCM, the better BCM performance. In our workflow, the evaluation step requires both BCM-corrected experiments and raw meta-experiment to evaluate the performance of BCMs and provides an output containing a list of each evaluation method applied to the input batch-corrected meta-experiment.

After assessing the performance of BCMs, we devised a summarised score based on each evaluation approach to rank each BCMs performance for the given meta-experiment. We describe the ranking step of selectBCM in the main manuscript in detail. We believe rank ordering of BCMs (**Diagnostic step**) will help users select top-performing BCMs for the given transcriptomic datasets and generate a diagnostic plot. We also provide a “**bcm_ranking**” function **to** provide the final rank of each BCM.

We offer an additional function named “**detect_batch**” using a subset of evaluation methods at the beginning of the workflow to detect batch effects in the merged meta-experiment obtained after the “**merge_experiment**” step to check whether the meta-experiment requires batch-correction or not.

**Supplementary text S4. A detailed description of osteoarthritis case study:**

To analyse the osteoarthritis (OA) datasets, we selected four experiments for osteoarthritis disease: E-GEOD-1919, E-GEOD-55235, E-MTAB-5564, and E-MTAB-51588. As expected, we observed that evaluation methods lacked consensus in choosing top-performing BCMs, and pcRegression was again least sensitive to the choice of BCM (**supplementary Fig 4A, supplementary Data D5**). In preserving biological information (disease parameter by PVCA and HVG.union), we observed that comBat2 and comBat1 ranked high among other BCMs. Based on overall performance, SelectBCM picked comBat2, Q_comBat, and comBat1 as the top 3 BCM (**supplementary Fig 4B**), which we analysed further.

We assess the impact of the top 3 BCMs for the OA meta-experiment similarly to the rheumatoid disease meta-analysis study. We observed that all 3 BCMs successfully mixed batches while improving the disease clustering (**supplementary Fig 4C**). For the OA meta-experiment, we obtained a similar set of degs after differential gene analysis(Q_comBat:645, comBat2:532 and comBat1:536). The metavolcanoR approach identified 438 degs perturbed across different datasets. We found 132 upregulated and 116 downregulated common across all methods (**supplementary Fig 4D)**. We found osteoarthritis-relevant processes such as platelet-derived growth factor binding[(7)](https://paperpile.com/c/qCQuVv/d4A21), protease binding[(8)](https://paperpile.com/c/qCQuVv/4Kr60), ER retention sequence binding[(9)](https://paperpile.com/c/qCQuVv/o3XhM) and calcium ion binding[(10)](https://paperpile.com/c/qCQuVv/3jHUF) as the top five upregulated molecular functions (**supplementary Fig 4E)**. Similarly, cytokine activity, protein homodimerisation activity, CXCR chemokine receptor binding, chemokine activity, and chemokine receptor binding were the top-five downregulated molecular functions relevant to OA disease biology (**supplementary Fig 4E)** [(11, 12)](https://paperpile.com/c/qCQuVv/EiNq1+KVUqx). Jensen tissue-specific enrichment of commonly upregulated genes also resulted in bone-related terms such as bone matrix, tendon, bone, myofibroblast and osteocytes. Therefore, we concluded that all three selected BCMs could infer osteoarthritis-related disease biology and agree with the MetavolcanoR approach for the OA meta-experiment.

**supplementary text S5. Macrophage activation assay**

For the selected macrophage activation assay, we limited our analysis to long exposure (18-24 hrs) to stimulants. We used macrophages differentiated with macrophage colony-stimulating factor (M-CSF) as control (M0) since it is the most commonly used method to generate monocyte-derived macrophages (MDMs) for both pro-and anti-inflammatory activations. We have the following proinflammatory (M1 phenotype) stimulants in our study design- lipopolysaccharides (LPS), interferon-γ (IFN-γ), tumour necrosis factor (TNF), interferon-α (IFN-α), toll-like receptor2/1 (TLR2/1) and, their combinations. The study includes two anti-inflammatories (M2 phenotype) stimulants interleukin-10 (IL10) and interleukin-4 (IL4).

**
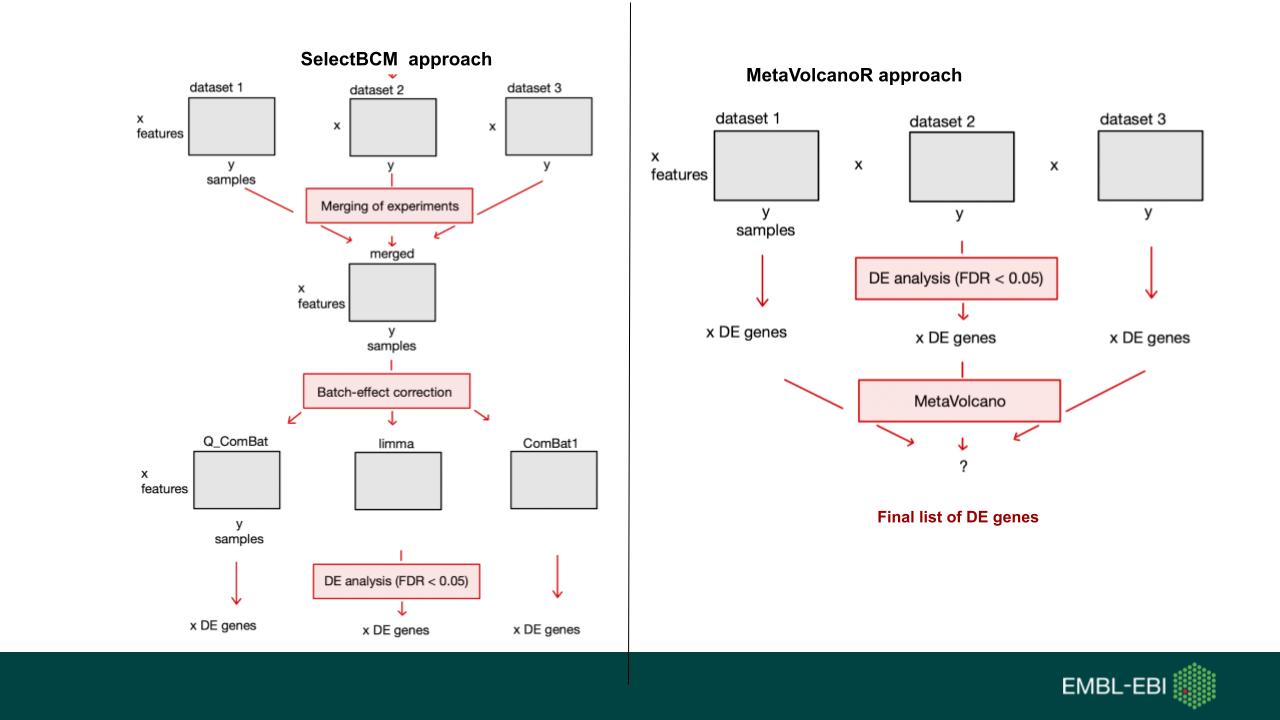
**

**Supplementary Fig 1.** Scheme to demonstrate differences between selectBCM and MetaVolcanoR approach for meta-analysis(Note: DE genes: differentially expressed genes).


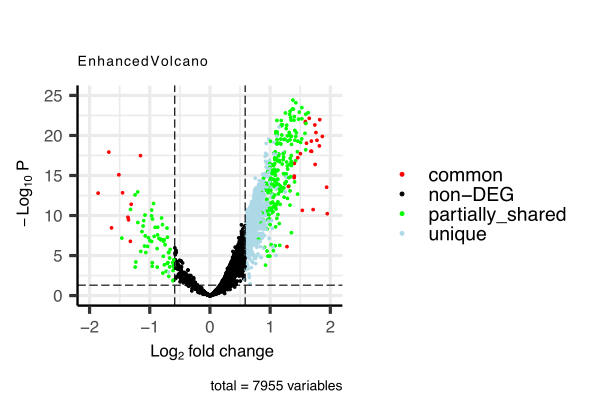
**Supplementary Fig 2.** Volcano plot of differentially expressed genes in rheumatoid arthritis meta-analysis. We investigate the reason for the discrepancy in the number of degs among different BCMs. We colour code DEGs based on whether they are shared among BCMs ( colour scheme: red: common DEGs among BCMs, green: partially shared limma DEGs, light blue: limma unique DEGs and black: non-DEGs). As illustrated in this plot, the unique DEGs (light blue) from limma have a strong tendency to show up in the low range of the fold-change. We also observed that the common and partially shared DEGs have the highest fold-change among other limma DEGs. Therefore, a stricter threshold, the fold-change cut-off of 1.5, is required for the limma method.

**
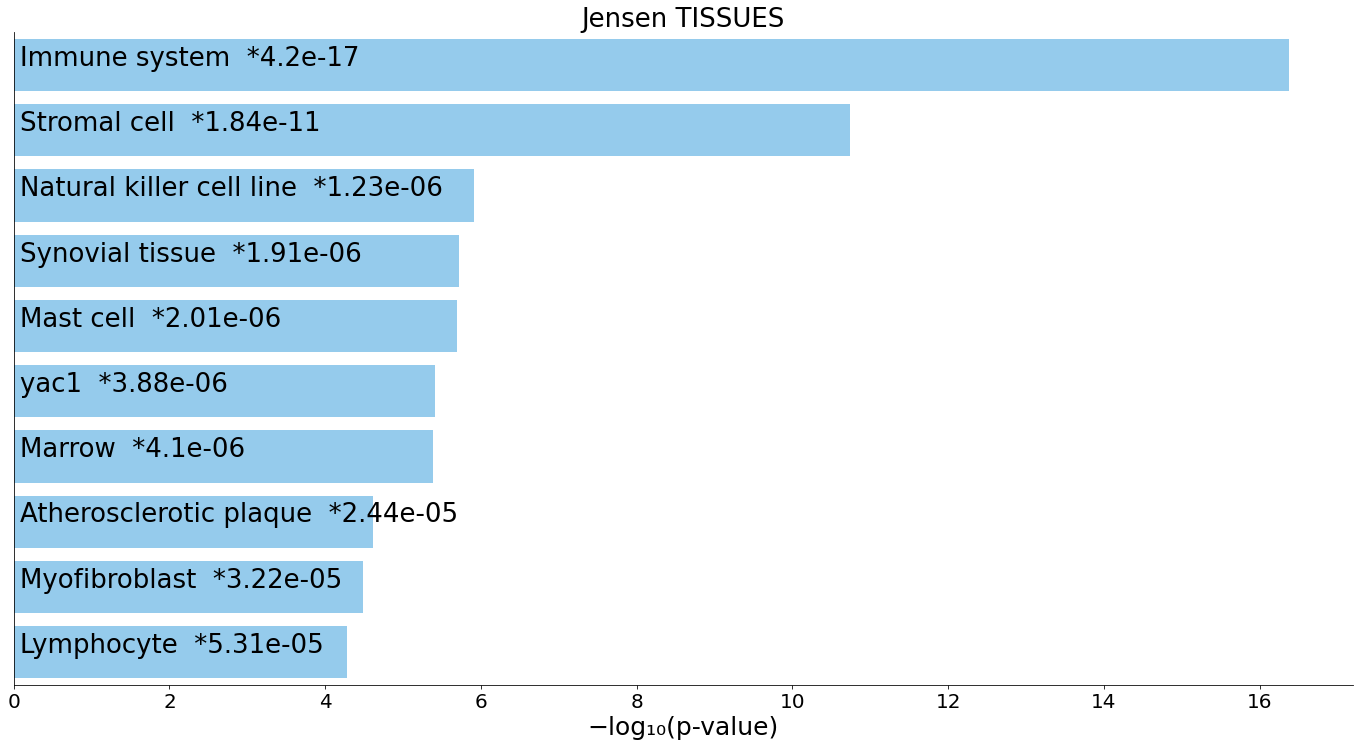
**

**Supplementary Fig 3.** Jensen tissue enrichment analysis of commonly upregulated genes in rheumatoid arthritis shows a strong immune response and synovial tissue enrichment.


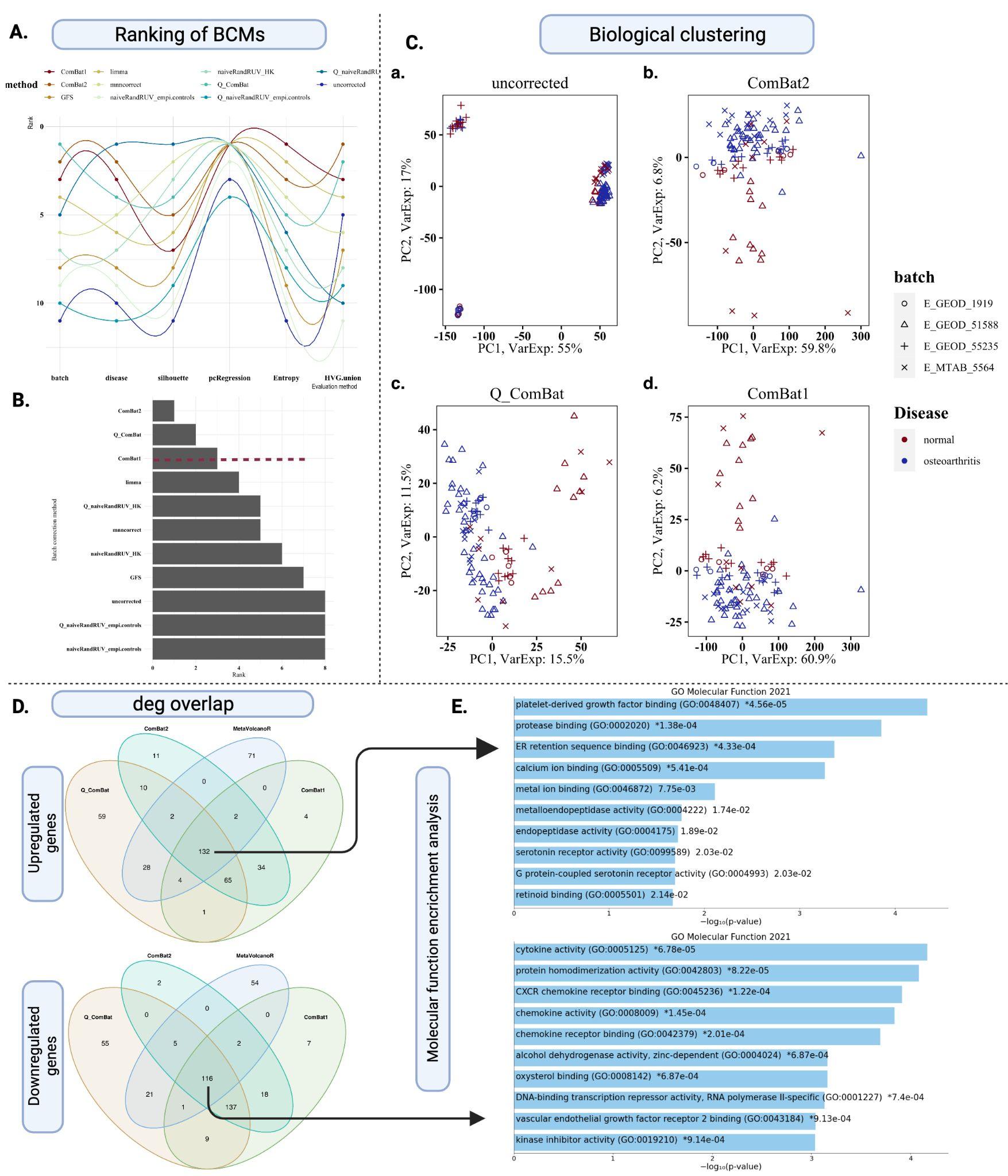


**Supplementary Fig 4. Meta-analysis of osteoarthritis(OA).** (A) The diagnostic plot shows an inconsistent ranking of BCM among evaluation methods. (B) Barplot of sumRank provided by selectBCM shortlists comBat2, Q_comBat and comBat1as top BCMs for the OA meta-experiment. (c) PCA plot shows that batch correction improves biological clustering; biosample colour is by disease class and shape by batch. (D) Venn diagram of the overlap of upregulated and downregulated genes among methods. (E) GO Molecular function enrichment analysis of common up and downregulated genes among top BCMs and the metaVolcanoR captured relevant disease biology.

**
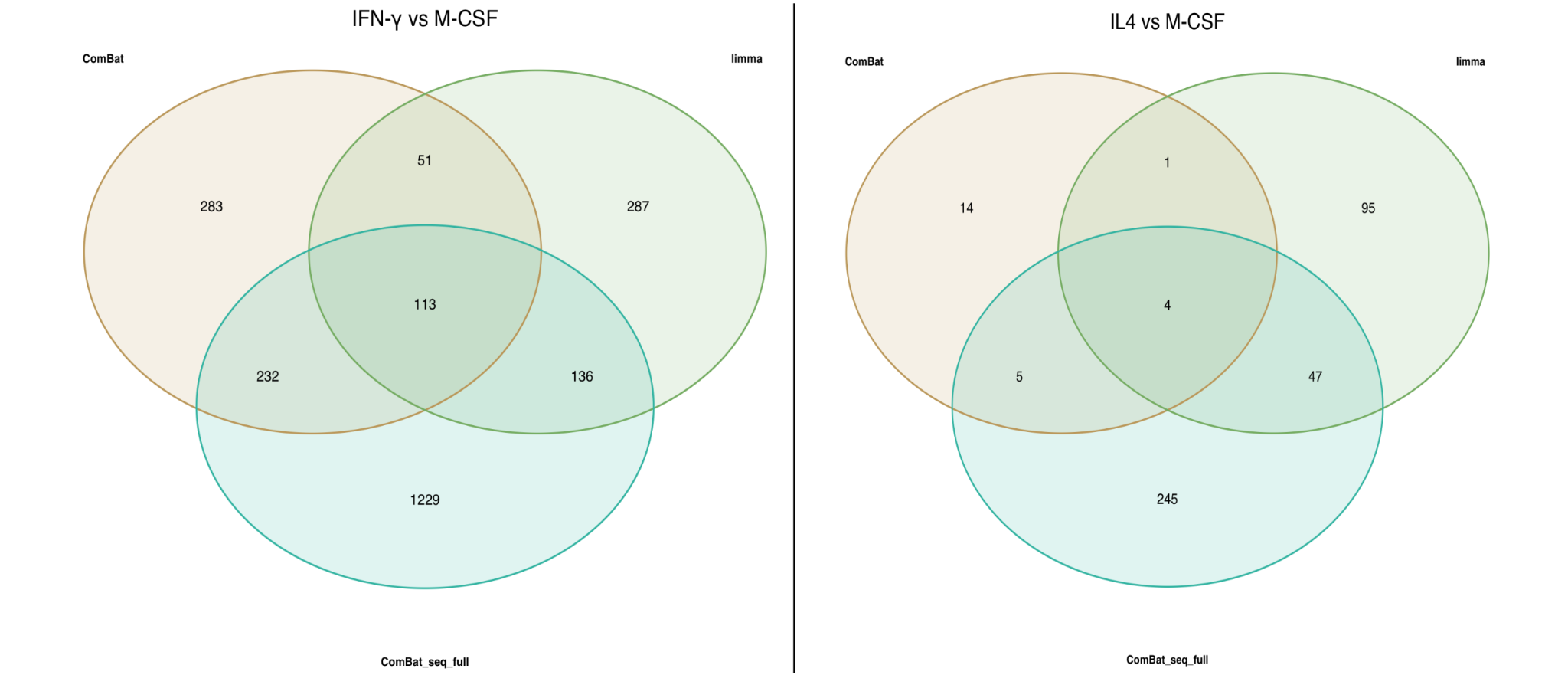
**

**Supplementary Fig 5.** Venn diagram of differentially expressed genes obtained from top 3 BCM corrected data for *in-vitro* MDMs meta-experiment.

**
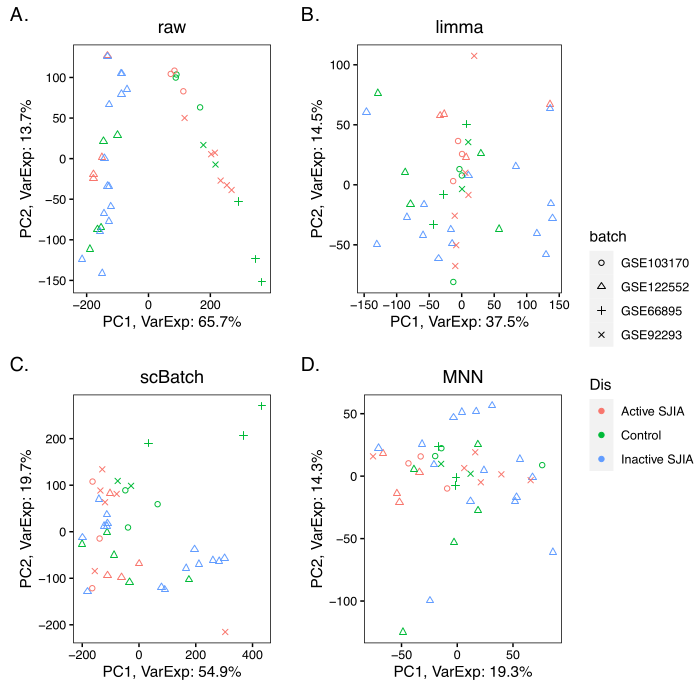
**

**Supplementary Fig 6.** In the case of neutrophile data for sJIA disease (dataset description: **Supplementary Data D7a,b)**, we observed that clustering did not improve after batch correction, which could be attributed to other disease classes or inherent biological heterogeneity or could be because of data-set imbalance. We further failed to identify any significant genes associated with the disease(at adj. p-value of 0.05, we did not identify any significant genes in any condition).

**
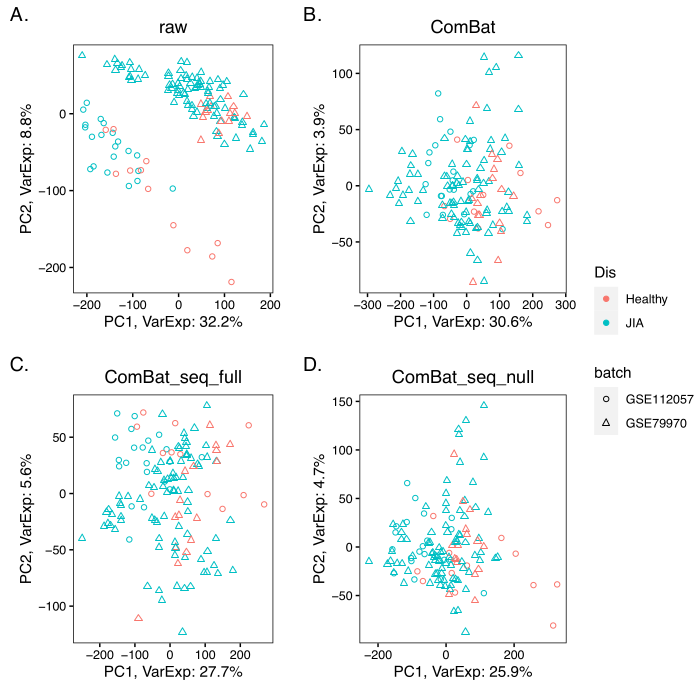
**

**Supplementary Fig 7.** In this test study, we chose only a subset of the whole blood/PBMC dataset belonging only to healthy or JIA and performed batch correction (**Supplementary Data D7a)**. Still, we observed that clustering did not improve after performing batch correction. We further failed to identify any significant genes associated with the disease( data not shown).

**
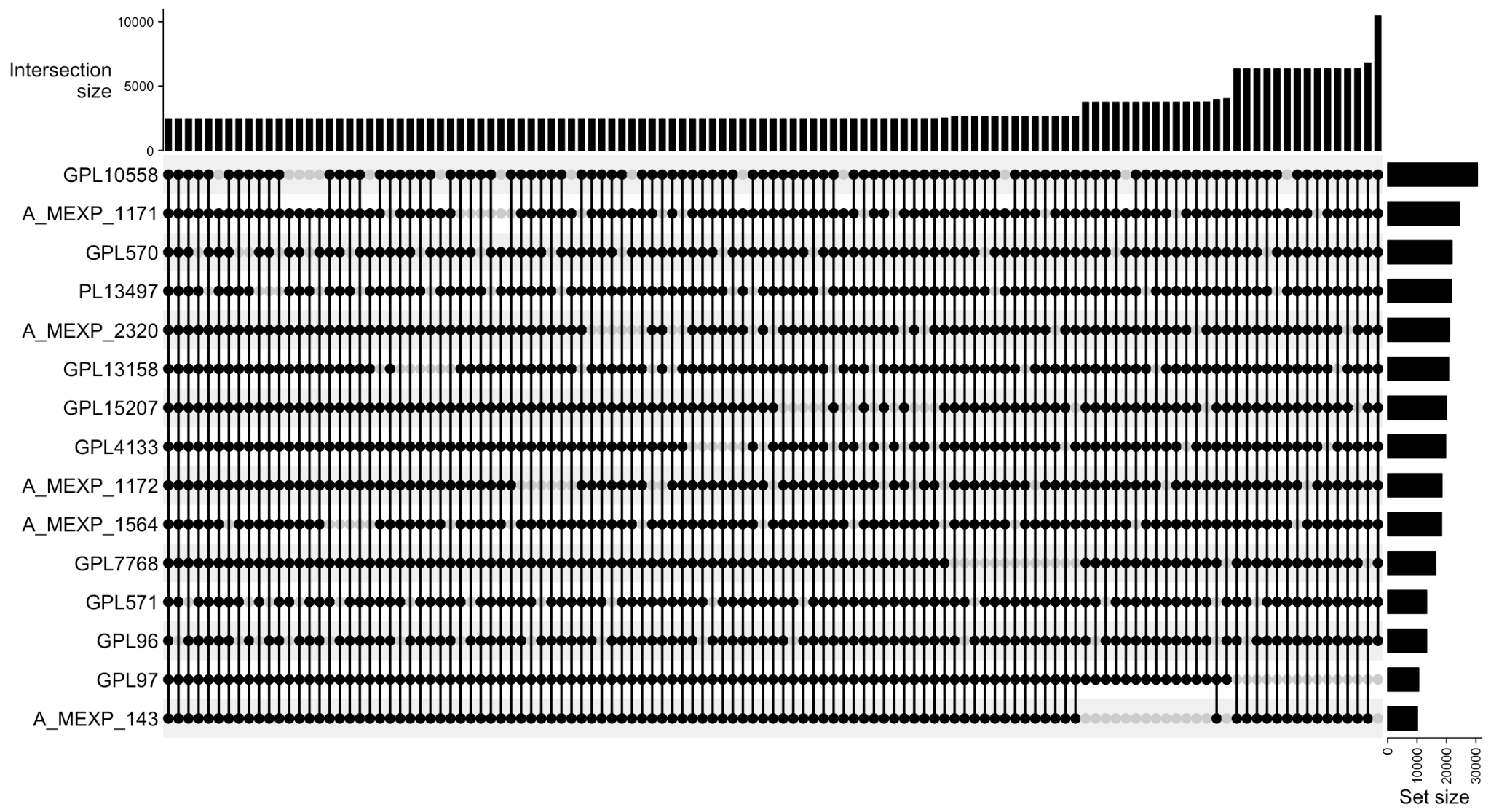
**

**Supplementary Fig 8. Array comparison.** We collected and compared array/chip information for 43 rheumatoid arthritis experiments. We observed that many chips were incompatible with others, and further data integration would result in less than 5000 genes to begin the analysis. Therefore, we strongly suggest users check array compatibility before starting a meta-analysis.

**Supplementary Data D1.** Summary of the list of implemented batch correction methods in the selectBCM tool.

| **Tool** | **Application** | **source /package** | **Technology** | **Input** | **output** | **useability** | **Ref** |
| --- | --- | --- | --- | --- | --- | --- | --- |
| removeBatchEffect | Batch-correction | limma | Microarray  /RNAseq | **Microarray**: log-expression matrix  **RNAseq**: count matrix | a numeric matrix of log-expression values with batch and covariate effects removed. | Data visualisation and unsupervised analyses such as PCA, MDS or heatmaps | [(13)](https://paperpile.com/c/qCQuVv/I4rO) |
| Gene Fuzzy Score (GFS) | Normalisation | Standalone | Microarray | **Microarray**: raw  expression matrix | Batch-corrected expression matrix | Various downstream analysis | [(14)](https://paperpile.com/c/qCQuVv/fXRV) |
| Robust quantile normalisation | Normalisation | PreprocessCore | Microarray | **Microarray**: log-expression matrix | A normalised gene-expression matrix | Various downstream analysis | [(15)](https://paperpile.com/c/qCQuVv/FgEd) |
| ComBat | Batch-correction | SVA | Microarray/RNAseq | **Microarray**: log-expression matrix  **RNAseq**: count matrix | a numeric matrix of log-expression values with batch and covariate effects removed. | Various downstream analysis | [(16)](https://paperpile.com/c/qCQuVv/2Zrn) |
| mnnCorrect | Batch-correction | Batchelor | Microarray/RNAseq | **Microarray**: log-expression matrix  **RNAseq**: count matrix | A normalised gene-expression matrix | Various downstream analysis | [(17)](https://paperpile.com/c/qCQuVv/tew7) |
| naiveRandRUV | Batch-correction | RUVnormalize | Microarray | **Microarray**: log-expression values matrix | A numeric matrix of log-expression values with batch and covariate effects removed. | Various downstream analysis | [(18)](https://paperpile.com/c/qCQuVv/W3W0) |
| ComBatSeq | Batch-correction | Standalone/git version | RNAseq | **RNAseq**: count matrix |  | Data visualisation and unsupervised analyses | [(19)](https://paperpile.com/c/qCQuVv/h08u) |
| scBatch | Batch-correction | http://github.com/tengfei-emory/scBatch | RNAseq | **RNAseq**: count matrix | A normalised count matrix | Various downstream analysis | [(20)](https://paperpile.com/c/qCQuVv/sbDu) |
| RUVs | Batch-correction | RUVSeq | RNAseq | **RNAseq**: count matrix | normalised count matrix | Various downstream analysis | [(21)](https://paperpile.com/c/qCQuVv/ry4k) |

**Supplementary Data D2.** Summary of the list of implemented evaluation methods in the selectBCM tool.

| **Evaluation methods** | **source /package** | **Input** | **output** |
| --- | --- | --- | --- |
| **Principal variance component analysis (PVCA)** | PVCA:  pvcaBatchAssess | 1. The Normalized gene-expression matrix (row: gene/feature, col: sample) 2. metadata matrix containing predictor variables | vector of proportions of variation explained by each predictor |
| **Silhouette coefficient** | MNN | 1. the principal components (PCs) 2. vector with the batch | average silhouette width for all clusters |
| **pcRegression** | κBET | Top 20 PCs | scaled pcRegression score |
| **Entropy** | MNN | regional entropy of batch mixing before and after batch correction using the first two PCs | BatchEntropy |
| **Highly variable genes (HVGs)** | la brennecke et al. 2019 | The Normalized gene-expression matrix (row: gene/feature, col: sample) | HVGs |

**Supplementary Data D3.** Summary of the experiments considered in a meta-analysis of rheumatoid arthritis and osteoarthritis study.

| **Experiment id** | **Description** | **tissue** | **platform** | **ref.** |
| --- | --- | --- | --- | --- |
| E-GEOD-1919 | Control, OA, RA | Synovial tissue | Affymetrix HG_U95A | [(22)](https://paperpile.com/c/qCQuVv/MMSC) |
| E-GEOD-55235 | Control, OA, RA | Synovial tissue | Affymetrix HG_U133A | [(23)](https://paperpile.com/c/qCQuVv/68MP) |
| E-MTAB-5564 | Control, OA | Bone | Agilent G3v2 | [(24)](https://paperpile.com/c/qCQuVv/cR2E) |
| E-GEOD-51588 | Control, OA | Subchondral Bone | Agilent Whole Human Genome Microarray 4x44K v2 | [(25)](https://paperpile.com/c/qCQuVv/Neh5) |
| E-GEOD-48780 | RA | Synovial tissue | Affymetrix HG_U133_Plus_2 | [(26)](https://paperpile.com/c/qCQuVv/Ww4D) |
| E-GEOD-12021 | Control, OA, RA | Synovial tissue | Affymetrix HG_U133A & Affymetrix HG_U133B | [(27)](https://paperpile.com/c/qCQuVv/5Ryv) |
| E-GEOD-55457 | Control, OA, RA | Synovial tissue | Affymetrix HG_U133A | [(23)](https://paperpile.com/c/qCQuVv/68MP) |
| E-MTAB-3201 | Control, psoriatic arthritis | PBMC/ Synovial tissue | Affymetrix HG_U133A_2 | [(24)](https://paperpile.com/c/qCQuVv/cR2E) |

**Note.**  we removed biosamples from diseases other than RA and OA from each experiment. Experiments with at least ten biosamples were only considered.

**Supplementary Data D4.** The output of the selectBCM tool to rank of BCMs for RA meta-analysis.

| **BCM** | **batch** | **disease** | **silhouette** | **pcRegression** | **Entropy** | **HVG.union** | **sumRank** |
| --- | --- | --- | --- | --- | --- | --- | --- |
| **limma** | **1** | **1** | **6** | **1** | **4** | **5** | **3** |
| GFS | 2 | 11 | 5 | 1 | 7 | 7 | 5 |
| **uncorrected** | **11** | **9** | **11** | **3** | **11** | **6** | **9** |
| **ComBat1** | **4** | **4** | **4** | **1** | **1** | **3** | **2** |
| ComBat2 | 5 | 2 | 1 | 1 | 5 | 4 | 3 |
| **Q_ComBat** | **3** | **3** | **2** | **1** | **2** | **2** | **1** |
| mnncorrect | 6 | 5 | 3 | 1 | 3 | 1 | 4 |
| Q_naiveRandRUV_HK | 7 | 6 | 7 | 2 | 9 | 10 | 7 |
| naiveRandRUV_HK | 8 | 7 | 8 | 1 | 6 | 9 | 6 |
| naiveRandRUV_empi.controls | 9 | 10 | 9 | 5 | 8 | 8 | 8 |
| Q_naiveRandRUV_empi.controls | 10 | 8 | 10 | 4 | 10 | 9 | 9 |

**Supplementary Data D5.** The output of the selectBCM tool to rank of BCMs for OA meta-analysis.

| **BCM** | **batch** | **disease** | **silhouette** | **pcRegression** | **Entropy** | **HVG.union** | **sumRank** |
| --- | --- | --- | --- | --- | --- | --- | --- |
| limma | 4 | 6 | 6 | 1 | 2 | 4 | 4 |
| GFS | 8 | 8 | 8 | 1 | 9 | 7 | 7 |
| uncorrected | 11 | 10 | 11 | 3 | 11 | 5 | 8 |
| **ComBat1** | **3** | **3** | **7** | **1** | **1** | **3** | **3** |
| **ComBat2** | **2** | **2** | **5** | **1** | **3** | **1** | **1** |
| **Q_ComBat** | **1** | **4** | **4** | **1** | **5** | **2** | **2** |
| mnncorrect | 6 | 5 | 2 | 1 | 4 | 6 | 5 |
| Q_naiveRandRUV_HK | 5 | 1 | 1 | 1 | 6 | 10 | 5 |
| naiveRandRUV_HK | 7 | 7 | 3 | 1 | 7 | 8 | 6 |
| naiveRandRUV_empi.controls | 9 | 9 | 10 | 2 | 10 | 11 | 8 |
| Q_naiveRandRUV_empi.controls | 10 | 11 | 9 | 4 | 8 | 9 | 8 |

**Supplementary Data D6.** The output of selectBCM tool to rank BCMs for macrophage activation assay meta-analysis.

| **BCM** | **batch** | **stimulant** | **silhouette** | **pcRegression** | **Entropy** | **HVG.union** | **sumRank** |
| --- | --- | --- | --- | --- | --- | --- | --- |
| ComBat_Seq_full | 2 | 1 | 2 | 3 | 2 | 4 | 1 |
| ComBat | 3 | 3 | 3 | 1 | 6 | 1 | 2 |
| limma | 1 | 2 | 5 | 2 | 3 | 7 | 3 |
| MNN | 4 | 4 | 1 | 1 | 4 | 9 | 4 |
| ComBat_seq_null | 5 | 5 | 4 | 4 | 1 | 5 | 5 |
| uncorrected1 | 6 | 7 | 8 | 7 | 8 | 3 | 6 |
| scBatch | 9 | 9 | 6 | 5 | 5 | 6 | 7 |
| uncorrected | 7 | 8 | 9 | 6 | 9 | 2 | 8 |
| RUVs | 8 | 6 | 7 | 8 | 7 | 8 | 9 |

**Supplementary Data D7a.** RNAseq experiments for meta-analysis of systemic Juvenile idiopathic arthritis (sJIA).

| **GSE ids** | **Cell_type** | **No. of samples** | **Disease** | **Control** | **Instrument** | **Processed data** |
| --- | --- | --- | --- | --- | --- | --- |
| GSE122552 | Neutrophil | 24 | 19 | 5 | Ion Torrent S5 | RPKM |
| GSE66895 | Neutrophil | 3 | - | 3 | Illumina HiSeq 2500 | RPKM |
| GSE92293 | Neutrophil | 8 | 6 | 2 | Illumina HiSeq 2000 | FPKM |
| GSE103170 | Neutrophil | 6 | 3 | 3 | NextSeq 500 | Raw count |
| GSE112057 | Whole blood | 175 | 91 | 12 | Illumina HiSeq 2000 | Raw count |
| GSE79970 | PBMC | 101 | 85 | 16 | Illumina HiSeq 2500 | Raw count |

**Supplementary Data D7b.** We summarise known biological heterogeneity in GSE122552 and GSE92293 datasets and the group imbalance present in GSE66895.

| **GSE ids** | **Cell_type** | **Biological heterogeneity** | **Disease** | **Control** |
| --- | --- | --- | --- | --- |
| GSE122552 | Neutrophil | Active and inactive state of SJIA | 19 | 5 |
| GSE66895 | Neutrophil | - | - | 3 |
| GSE92293 | Neutrophil | 3 ADU (active disease, untreated), 3 ADT (active disease, treated) and 2 HC (healthy control) | 6 | 2 |
| GSE103170 | Neutrophil | Active | 3 | 3 |

**Note.** For analysis, gene-level expression counts were generated by the STAR alignment method, and only QC-passed samples were used. We analysed neutrophil and whole blood/PBMC data separately. We are showing results separately for neutrophil and whole blood/PBMC data.

**Additional Bibliography**

[1. Hochberg,Y. and Benjamini,Y. (1990) More powerful procedures for multiple significance testing. *Statistics in Medicine*, **9**, 811–818.](http://paperpile.com/b/qCQuVv/4yqd)

[2. Xie,Z., Bailey,A., Kuleshov,M.V., Clarke,D.J.B., Evangelista,J.E., Jenkins,S.L., Lachmann,A., Wojciechowicz,M.L., Kropiwnicki,E., Jagodnik,K.M., *et al.* (2021) Gene Set Knowledge Discovery with Enrichr. *Curr Protoc*, **1**, e90.](http://paperpile.com/b/qCQuVv/mucc)

[3. Ashburner,M., Ball,C.A., Blake,J.A., Botstein,D., Butler,H., Cherry,J.M., Davis,A.P., Dolinski,K., Dwight,S.S., Eppig,J.T., *et al.* (2000) Gene ontology: tool for the unification of biology. The Gene Ontology Consortium. *Nat. Genet.*, **25**, 25–29.](http://paperpile.com/b/qCQuVv/zrKV)

[4. Palasca,O., Santos,A., Stolte,C., Gorodkin,J. and Jensen,L.J. (2018) TISSUES 2.0: an integrative web resource on mammalian tissue expression. *Database*, **2018**.](http://paperpile.com/b/qCQuVv/1FBl)

[5. MetaVolcanoR *Bioconductor*.](http://paperpile.com/b/qCQuVv/VAcA)

[6. Prada-Medina,C.A., Peron,J.P.S. and Nakaya,H.I. (2020) Immature neutrophil signature associated with the sexual dimorphism of systemic juvenile idiopathic arthritis. *J. Leukoc. Biol.*, **108**, 1319–1327.](http://paperpile.com/b/qCQuVv/jOfq)

[7. Setiawan,I.G.N.Y., Suyasa,I.K., Astawa,P., Dusak,I.W.S., Kawiyana,I.K.S. and Aryana,I.G.N.W. (2019) Recombinant platelet derived growth factor-BB and hyaluronic acid effect in rat osteoarthritis models. *J Orthop*, **16**, 230–233.](http://paperpile.com/b/qCQuVv/d4A21)

[8. Troeberg,L. and Nagase,H. (2012) Proteases involved in cartilage matrix degradation in osteoarthritis. *Biochim. Biophys. Acta*, **1824**, 133–145.](http://paperpile.com/b/qCQuVv/4Kr60)

[9. Hughes,A., Oxford,A.E., Tawara,K., Jorcyk,C.L. and Oxford,J.T. (2017) Endoplasmic Reticulum Stress and Unfolded Protein Response in Cartilage Pathophysiology; Contributing Factors to Apoptosis and Osteoarthritis. *Int. J. Mol. Sci.*, **18**.](http://paperpile.com/b/qCQuVv/o3XhM)

[10. Fan,X., Wu,X., De Lima,L.T.F., Stehbens,S., Punyadeera,C., Webb,R., Hamilton,B., Ayyapann,V., McLauchlan,C., Crawford,R., *et al.* (2022) The deterioration of calcified cartilage integrity reflects the severity of osteoarthritis—A structural, molecular, and biochemical analysis. *The FASEB Journal*, **36**.](http://paperpile.com/b/qCQuVv/3jHUF)

[11. Scanzello,C.R. (2017) Chemokines and inflammation in osteoarthritis: Insights from patients and animal models. *J. Orthop. Res.*, **35**, 735–739.](http://paperpile.com/b/qCQuVv/EiNq1)

[12. Endres,M., Andreas,K., Kalwitz,G., Freymann,U., Neumann,K., Ringe,J., Sittinger,M., Häupl,T. and Kaps,C. (2010) Chemokine profile of synovial fluid from normal, osteoarthritis and rheumatoid arthritis patients: CCL25, CXCL10 and XCL1 recruit human subchondral mesenchymal progenitor cells. *Osteoarthritis Cartilage*, **18**, 1458–1466.](http://paperpile.com/b/qCQuVv/KVUqx)

[13. Ritchie,M.E., Phipson,B., Wu,D., Hu,Y., Law,C.W., Shi,W. and Smyth,G.K. (2015) limma powers differential expression analyses for RNA-sequencing and microarray studies. *Nucleic Acids Res.*, **43**, e47.](http://paperpile.com/b/qCQuVv/I4rO)

[14. Belorkar,A. and Wong,L. (2016) GFS: fuzzy preprocessing for effective gene expression analysis. *BMC Bioinformatics*, **17**, 540.](http://paperpile.com/b/qCQuVv/fXRV)

[15. bmbolstad.com>,B.B. <bmb at (2022) preprocessCore: A collection of pre-processing functions Bioconductor version: Release (3.15).](http://paperpile.com/b/qCQuVv/FgEd)

[16. Leek,J.T., Johnson,W.E., Parker,H.S., Jaffe,A.E. and Storey,J.D. (2012) The sva package for removing batch effects and other unwanted variation in high-throughput experiments. *Bioinformatics*, **28**, 882–883.](http://paperpile.com/b/qCQuVv/2Zrn)

[17. Haghverdi,L., Lun,A.T.L., Morgan,M.D. and Marioni,J.C. (5/2018) Batch effects in single-cell RNA-sequencing data are corrected by matching mutual nearest neighbors. *Nat. Biotechnol.*, **36**, 421–427.](http://paperpile.com/b/qCQuVv/tew7)

[18. Jacob,L., Gagnon-Bartsch,J.A. and Speed,T.P. (2016) Correcting gene expression data when neither the unwanted variation nor the factor of interest are observed. *Biostatistics*, **17**, 16–28.](http://paperpile.com/b/qCQuVv/W3W0)

[19. Zhang,Y., Parmigiani,G. and Johnson,W.E. (2020) ComBat-seq: batch effect adjustment for RNA-seq count data. *NAR Genomics and Bioinformatics*, **2**, lqaa078.](http://paperpile.com/b/qCQuVv/h08u)

[20. Fei,T. and Yu,T. (2020) scBatch: batch-effect correction of RNA-seq data through sample distance matrix adjustment. *Bioinformatics*, **36**, 3115–3123.](http://paperpile.com/b/qCQuVv/sbDu)

[21. Risso,D., Ngai,J., Speed,T.P. and Dudoit,S. (2014) Normalization of RNA-seq data using factor analysis of control genes or samples. *Nat. Biotechnol.*, **32**, 896–902.](http://paperpile.com/b/qCQuVv/ry4k)

[22. Ungethuem,U., Haeupl,T., Witt,H., Koczan,D., Krenn,V., Huber,H., von Helversen,T.M., Drungowski,M., Seyfert,C., Zacher,J., *et al.* (2010) Molecular signatures and new candidates to target the pathogenesis of rheumatoid arthritis. *Physiol. Genomics*, **42A**, 267–282.](http://paperpile.com/b/qCQuVv/MMSC)

[23. Woetzel,D., Huber,R., Kupfer,P., Pohlers,D., Pfaff,M., Driesch,D., Häupl,T., Koczan,D., Stiehl,P., Guthke,R., *et al.* (2014) Identification of rheumatoid arthritis and osteoarthritis patients by transcriptome-based rule set generation. *Arthritis Res. Ther.*, **16**, R84.](http://paperpile.com/b/qCQuVv/68MP)

[24. Athar,A., Füllgrabe,A., George,N., Iqbal,H., Huerta,L., Ali,A., Snow,C., Fonseca,N.A., Petryszak,R., Papatheodorou,I., *et al.* (2019) ArrayExpress update - from bulk to single-cell expression data. *Nucleic Acids Res.*, **47**, D711–D715.](http://paperpile.com/b/qCQuVv/cR2E)

[25. Chou,C.-H., Wu,C.-C., Song,I.-W., Chuang,H.-P., Lu,L.-S., Chang,J.-H., Kuo,S.-Y., Lee,C.-H., Wu,J.-Y., Chen,Y.-T., *et al.* (2013) Genome-wide expression profiles of subchondral bone in osteoarthritis. *Arthritis Res. Ther.*, **15**, R190.](http://paperpile.com/b/qCQuVv/Neh5)

[26. Sun,Y., Caplazi,P., Zhang,J., Mazloom,A., Kummerfeld,S., Quinones,G., Senger,K., Lesch,J., Peng,I., Sebrell,A., *et al.* (2014) PILRα negatively regulates mouse inflammatory arthritis. *J. Immunol.*, **193**, 860–870.](http://paperpile.com/b/qCQuVv/Ww4D)

[27. Huber,R., Hummert,C., Gausmann,U., Pohlers,D., Koczan,D., Guthke,R. and Kinne,R.W. (2008) Identification of intra-group, inter-individual, and gene-specific variances in mRNA expression profiles in the rheumatoid arthritis synovial membrane. *Arthritis Res. Ther.*, **10**, R98.](http://paperpile.com/b/qCQuVv/5Ryv)
